# Short-Term Intake of Chlorogenic Acids Improves Psychomotor Speed and Motor Speed in Adults: A Randomized Crossover Trial

**DOI:** 10.3390/brainsci12030370

**Published:** 2022-03-11

**Authors:** Chika Suzukamo, Ryuji Ochiai, Yuki Mitsui, Noriko Osaki, Takahiro Ono

**Affiliations:** 1 Biological Science Research, Kao Corporation, Tokyo 131-8501, Japan; suzukamo.chika@kao.com (C.S.); mitsui.yuki@kao.com (Y.M.); 2 Health & Wellness Products Research, Kao Corporation, Tokyo 131-8501, Japan; osaki.noriko@kao.com; 3 Ueno-Asagao Clinic, Kairaku Building 6F, Tokyo 110-0015, Japan; info@ueno-asagao.clinic

**Keywords:** chlorogenic acids, cognitive function, randomized controlled trial, psychomotor speed, motor speed

## Abstract

Chlorogenic acid (CGA), a polyphenolic compound found in various plants, has been reported to improve cognitive function. However, it remains unclear how long it takes for CGAs to exert their effects. Here, we evaluated the short-term effects of CGAs on cognitive function. We assessed the effects of 2-week CGA intake on cognitive function. The study was carried out as a randomized, double-blind, placebo-controlled, crossover trial. Twenty-six healthy Japanese participants (50–65 years of age) were randomly assigned to either the active beverage (CGAs: 270 mg) or the placebo beverage group daily for 2 weeks. After a 2-week washout period, the participants consumed the other beverages. We assessed cognitive function at baseline and following the first treatment period using the Japanese version of CNS Vital Signs. CGAs significantly improved the scores for psychomotor speed, motor speed, and right and left finger tapping compared to placebo. In addition, processing speed scores improved significantly from baseline only after CGA intake. In conclusion, CGAs were confirmed to improve cognitive function over a short period of two weeks.

## 1. Introduction

Cognitive function encompasses abilities such as memory, attention, language, and executive function; most of these decline with age [1]. Cognitive impairments hinder the independence of older adults and reduce their quality of life (QOL) [2]. By 2050, approximately 16% of the global population will be estimated to be 65 years or older [3]. Thus, maintaining cognitive function in older adults is an important global issue. Lifestyle habits such as exercise, diet, and psychosocial stress affect the maintenance of cognitive function [4].

Various polyphenol compounds have many health benefits and have shown their therapeutic potential in the treatment of cognitive impairment [5]. CGAs (i.e., caffeoylquinic acids, feruloylquinic acids, and dicaffeoylquinic acids) are polyphenolic compounds found in various plants and are particularly abundant in green coffee beans [6]. CGAs and their metabolites have been shown to have many benefits, such as improving insulin resistance and hypertension [7,8], sleep quality and fatigue [9], incurring neuroprotective effects against oxidative stress [10], and promoting neuronal differentiation [11]. Furthermore, CGAs have been shown to prevent cognitive deficits in a mouse model of Alzheimer’s disease [12].

Several human studies have investigated the effects of continuous CGA intake on cognitive function. For example, taking CGAs daily for four to six months improved the cognitive function of middle-aged and older adults [13,14], and daily intake for three months improved attention and executive function in individuals with mild cognitive impairment (MCI) [15]. In contrast, the effects of acute CGA intake had a limited impact, if any, on cognitive function [16]. Therefore, we sought to verify how long it takes CGAs to affect cognitive function. To date, two weeks of daily CGA consumption has been shown to improve sleep quality and fatigue [9], vascular endothelial function [17], and systolic blood pressure [18]. Thus, a 2-week consistent CGA intake may also have beneficial effects on cognitive function.

In this study, we aimed to evaluate the effects of short-term CGAs, the main components of coffee polyphenols, on cognitive function and assess how long it takes for CGAs to improve cognitive function. Given that the minimum period in which the benefits of CGAs could be confirmed is two weeks, we investigated the effects of 2-week CGA intake on cognitive function.

## 2. Materials and Methods

### 2.1. Participants

Volunteers who were aware of their cognitive decline, but were otherwise healthy and living independently, were recruited from the Tokyo metropolitan area. The eligibility criteria were as follows: healthy participants, 50–65 years of age, aware of their cognitive decline, without any difficulty in dealing with a personal computer, without any signs of color blindness, and with “psychomotor speed” scores, as evaluated using the Japanese version of CNS Vital Signs (CNSVS; CNS Vital Signs, LLC., Morrisville, NC, USA), known as “Cognitrax” in Japan, falling below average values. The exclusion criteria were as follows: MCI or dementia, food allergies, a history of serious disease, excessive alcohol or coffee intake, obesity, smoking, color vision disability, difficulty using computers, participation in another clinical trial, taking medicines or supplements that might influence cognitive function, and disqualification by the physician in charge. Written informed consent was obtained from all participants prior to the start of the study. This is the first trial on the effects of short-term ingestion of chlorogenic acids, and the sample size (12 per group) was estimated based on Julious’ method [19]. To account for potential dropouts, the target number of participants was set to 13 per group.

### 2.2. Materials

CGAs, the main components of coffee polyphenols, were extracted from Coffea canephora green coffee beans using a hot-water extraction method [20]. The extract was filtered to reduce caffeine levels below the limit of quantification (<1 mg/100 g) to prevent its potential effects on cognitive function [21]. The filtered extract was then spray-dried to obtain a dry powder. CGAs in the extract comprised three types of compounds: caffeoylquinic acids (CQAs), feruloylquinic acids (FQAs), and dicaffeoylquinic acids (diCQAs). The composition of these CGA types, as assessed by high-performance liquid chromatography, was as follows: 70.2% CQAs, 13.4% FQAs, and 16.4% diCQAs.

The CGA-containing beverage was prepared using coffee bean extract, water, acidifiers, amino acids, vitamins, sweeteners, and flavoring agents. The total amount of CQAs and FQAs was 270 mg. The placebo beverage was identical, except that it contained no CGAs.

During the study period, participants ingested either the CGA-containing beverage or placebo daily, 30–60 min before bedtime.

### 2.3. Experimental Design

**Figure 1:**
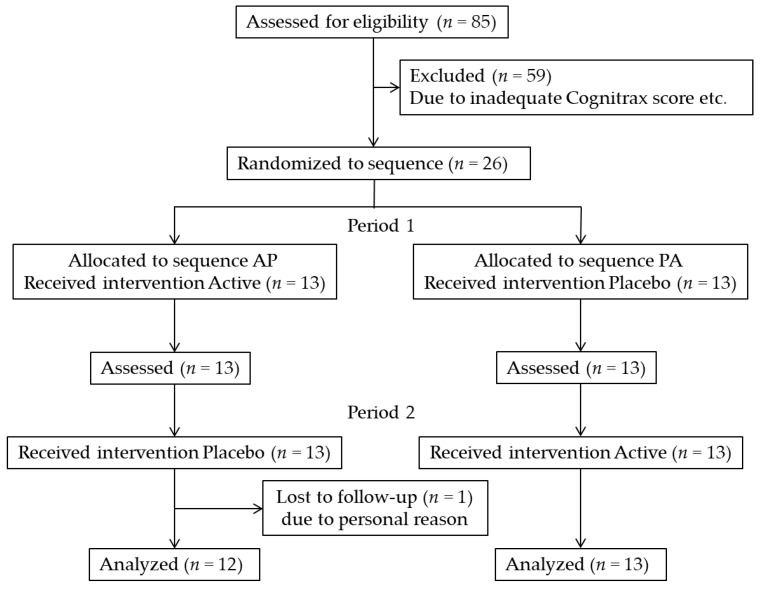
Flow diagram of the trial.

This was a randomized, double-blind, placebo-controlled, crossover trial study with a 2-week washout period between 2-week intervention periods. Participants who met the inclusion criteria were assigned to either sequence AP (active beverage followed by placebo beverage) or PA (placebo beverage followed by active beverage) (Figure 1). The allocation table was sealed until the trial was completed. During the intervention period, they ingested the active (270 mg) or placebo beverage for 2 weeks. Following the 2-week washout period, participants ingested the other beverage. The first 2-week intake period was referred to as Period 1, and the second 2-week intake period was referred to as Period 2. During the test period, participants were instructed to maintain their usual dietary habits, and the primary endpoint was cognitive function as assessed by Cognitrax.

This study was conducted at the Ueno-Asagao Clinic (Tokyo, Japan) from 1 September 2018 to 19 March 2019, and complied with the principles set forth in the Declaration of Helsinki (2013). The study was managed by a contract research organization (TES Holdings Corporation, Tokyo, Japan). All protocols were approved by the Ethics Committee of the Oriental Ueno Detection Center, General Incorporated Association Oriental Occupational Health Association Tokyo Branch (approval date: 30 July 2018), and the Human Research Ethics Committee of Kao Corporation (approval date: 19 July 2018). This trial was registered with the UMIN Clinical Trial Registry before enrollment of the first participant (UMIN ID: UMIN000033810).

### 2.4. Cognitive Function Assessment

Cognitrax (CNSVS) was used to evaluate cognitive function before treatment (baseline) and immediately after CGA or placebo treatment (2 W). The CNSVS is a computer-administered neurocognitive test battery with confirmed validity and reliability [22,23]. We used all seven tests from this battery of tests—the Verbal Memory Test (VBM), to investigate the ability of learning words, memory for words, word recognition, and immediate and delayed recall; the Visual Memory Test (VIM), to investigate the ability of learning shapes, memory for shapes, shapes recognition, and immediate and delayed recall; the Finger Tapping Test (FTT), to investigate the ability of motor speed and fine motor control; the Symbol Digit Test (SDC), to investigate the complex information processing accuracy, complex attention, visual-perceptual speed, and information processing speed; the Stroop Test (ST), to investigate the ability of simple reaction time, complex reaction time, Stroop reaction time, inhibition/disinhibition, and frontal or executive skills; the Shifting Attention Test (SAT), to investigate the ability of executive function, shifting sets (rules, categories, and rapid decision making), and reaction time; and the Continuous Performance Test (CPT), to investigate the ability to maintain sustained attention, choice rection time, and impulsivity.

It took approximately 30 min to complete all of the tests. Based on the scores of the seven tests, the composite neurocognitive index (NCI) and cognitive domain scores were generated (Table 1). All test and domain scores were presented as age-adjusted standard scores, with a mean of 100 and standard deviation (SD) of 15. Higher scores reflect higher levels of cognitive performance.

### 2.5. Statistical Analysis

Data are presented as the mean ± standard error (SE), unless otherwise indicated. All data for the two treatments were compared using a paired *t*-test (two-tailed, α = 0.05). The Wilcoxon signed-rank test was used when the distribution was not normal. Statistical significance was set at *p* < 0.05 (two-tailed). SPSS version 24 (IBM SPSS Statistics, IBM Corp., Tokyo, Japan) was used for all statistical analyses.

## 3. Results

### 3.1. Participants and Baseline Characteristics

Figure 1 represents the flow of participant selection. Of the 85 participants assessed for eligibility, 26 were enrolled and randomly assigned to one of the two treatment groups (sequence AP or PA; 13 participants per group). During Period 2, one participant (placebo beverage) withdrew from the study for personal reasons. In total, 25 participants (Sequence AP, *n* = 12; Sequence PA, *n* = 13) completed the trial by complying with the study protocol, which required consuming more than 80% of the test beverage. Data from these 25 participants were assessed. The baseline characteristics of the enrolled participants are presented in Table 2. Throughout the study period, there were no adverse events related to the consumption of the test beverages.

### 3.2. Participants and Baseline Characteristics

Table 3 represents the scores for individual tests in the CNSVS. There was a significant difference in the scores for right tap average and left tap average in the FTT between CGA intake and placebo. For CGA intake, there was a significant increase relative to baseline in scores of average and left tap average in the FTT, correct responses in the SDC, and correct reaction time in the SAT.

Table 4 represents the NCI and cognitive domain scores of the CNSVS. There was a significant difference in scores between CGA intake and placebo for psychomotor and motor speeds. With CGA intake, the scores of psychomotor speed and processing speed significantly improved from baseline, and with placebo, the score for composite memory significantly increased from baseline.

## 4. Discussion

In this study, we found that the period required to achieve the effects of CGAs, the main components of coffee polyphenols, on cognitive function for effectiveness lasted a total of two weeks. Although the effect is mild compared to long-term intake [13,14], the short-term intake of CGAs has also been shown to improve cognitive function. Among cognitive domain scores, psychomotor speed and motor speed were significantly increased with CGA intake compared to the placebo, which is consistent with previous studies of long-term intake. The psychomotor speed domain of the Cognitrax reflects how well an individual perceives, attends, and responds to visual-perceptual information and performs motor speed and fine motor coordination [23]. Motor speed forms a part of psychomotor speed. Psychomotor speed declines with age [24], in a manner similar to language, attention, and executive function. Moreover, low psychomotor speed in older adults is reportedly related to an increased risk of developing various brain disorders, such as dementia, Alzheimer’s disease, Parkinson’s disease, and depressive symptoms [25]. Therefore, improving psychomotor speed may also be beneficial as it attenuates dementia progression.

The psychomotor speed score was calculated using the FTT and SDC. For the FTT, the right and left tap averages significantly improved with CGA intake as compared to placebo. For the SDC, the number of correct responses was significantly increased relative to the baseline only after CGA intake. Improvements in the FTT score are consistent with previous reports [13,14], suggesting that CGAs have a particularly strong effect on FTT. The FTT is used to measure self-directed manual motor speed [26] and represents one of the most commonly used tests in neuropsychology given its simplicity and reliability [22] The FTT has historically been considered as a cerebellar test for evaluating dexterity [27]. Although dexterity is often thought to be related to fingertip movements, joint movements (e.g., wrist and elbow) are also important for dexterity. In recent years, research has progressed, elucidating the relationship between dexterity and the brain. According to one study, FTT activates the frontal lobe of the cerebral cortex, with marked activation observed in the supplementary motor area [28]. Thus, improvements in the FTT by CGA intake may reflect improvements not only in the cerebellum, but also in the frontal lobe. Moreover, reduced FTT scores have been reported in patients with MCI and AD [29]. Since the risk of falls increases with MCI, and atrophy has been reported in the supplementary motor area and anterior cingulate cortex in patients with MCI who experience falls [30], motor control and the frontal lobe are likely to be interrelated. Here, CGAs also improved scores of the SAT and SDC (tests that reflect frontal lobe function), as well as cognitive domain processing speed scores relative to baseline, further supporting the possibility that CGAs act on the frontal lobe to improve FTT scores. This possibility is also supported by improvements in frontal lobe function resulting from long-term CGA intake reported in previous studies [13,14,15]. Only a few domains in Cognitrax were improved with 2-week CGA intake in this study, so a longer period may be needed to improve other cognitive domains.

While CGAs have been shown to have neuroprotective effects [10] and promote neuronal differentiation [11] in vitro, the mechanisms underlying the improvements in the cognitive function of humans conferred by CGAs remain unknown. One possibility is that CGA intake contributes to improvements in vascular endothelial function. Vascular endothelial function has been reported to improve during a 2-week CGA intake period, likely by increasing the bioavailability of nitric oxide (NO) [17]. By improving the bioavailability of NO, CGAs may contribute to adequate cerebral blood flow, which in turn improves cognitive function. Moreover, the beneficial effects of CGAs on fatigue and sleep quality may have contributed to the improvements in cognitive function observed in the present trial. CGA intake over the course of two weeks has been reported to improve sleep quality and reduce fatigue [9]. Fatigue is an important clinical marker of cognitive deficits (including psychomotor speed) in non-demented older adults [31], suggesting that CGAs may improve cognitive function by reducing fatigue. While the above-mentioned indirect mechanisms may be involved in the effects of the short 2-week intake period, direct effects on the brain, such as the inhibition of amyloid β (Aβ) accumulation [12] or the suppression of neuronal death, may also be relevant to long-term CGA intake effects. In this study, 2-week intake of CGAs improved cognitive function, similarly to the long-term intake of CGAs. This could contribute to elucidating the mechanism underlying the improvement in cognitive function by the long-term intake of CGAs. Consistent with previous studies providing potential therapeutic effects of polyphenols [5], CGAs have been shown to have potential in the treatment of cognitive impairment. More studies are needed to clarify the optimal dose and period, as well as the pharmacokinetic profile.

This study has several limitations. First, we did not collect data on lifestyle rhythms, such as dietary or sleep patterns. The effects of CGAs on cognitive function and sleep need to be verified at the same time. However, we requested that participants maintain their normal lifestyle patterns during the study period. Second, the mechanisms underlying the observed improvements in cognitive function remain unclear. In future studies, it will be important to assess whether changes occur in brain activity. Third, it remains unclear from our findings whether the speed improvements of both cognitive function and movement observed that were observed with short-term CGA intake would provide a benefit against cognitive decline in the long term. Given that cognitive decline progresses over the course of several years, trials with long-term CGA intake are warranted.

In conclusion, this study probed the effects of a 2-week intervention with CGAs, the main components of coffee polyphenols, on cognitive function in middle-aged adults. CGAs improved psychomotor and motor speed, as assessed by a battery of tests, suggesting that CGAs are able to improve certain cognitive functions.

## Figures and Tables

**Table 1:** Cognitive domain scores in CNS Vital Signs.

Cognitive Domains	Tests for Score Calculations
Neurocognitive Index (NCI)	All of the tests
Composite Memory	VBM + VIM
Verbal Memory	VBM
Visual Memory	VIM
Psychomotor Speed	FTT + SDC
Reaction Time	ST
Complex Attention	ST + SAT + CPT
Cognitive Flexibility	ST + SAT
Processing Speed	SDC
Executive Function	SAT
Simple Attention	CPT
Motor Speed	FTT

VBM, Verbal Memory Test; VIM, Visual Memory Test; FTT, Finger Tapping Test; SDC, Symbol Digit Coding; ST, Stroop Test; SAT, Shifting Attention Test; CPT, Continuous Performance Test.

**Table 2:** Participant characteristics.

Characteristics	Mean ± SD
Number of subjects (male)	26 (12)
Age (y)	55.8 ± 2.8
Height (cm)	165.1 ± 8.6
Body weight (kg)	59.1 ± 12.3
Body fat (%)	24.0 ± 8.2
BMI (kg/m^2^)	21.6 ± 3.8
SBP (mmHg)	108.7 ± 14.5
DBP (mmHg)	67.9 ± 10.0

BMI, body mass index; SBP, systolic blood pressure; DBP, diastolic blood pressure.

**Table 3:** The standardized scores of individual tests in Cognitrax.

		Group	Baseline	2W	*p*-Value
Verbal Memory Test (VBM)	Correct Hits (Immediate)	CGA	90.0 ± 3.1	93.9 ± 2.5	*p* = 0.981
		Placebo		93.8 ± 2.0	
	Correct Passes (Immediate)	CGA	103.4 ± 2.4	102.3 ± 2.8	*p* = 0.281
		Placebo		105.0 ± 1.8	
	Correct Hits (Deley)	CGA	95.6 ± 3.6	97.2 ± 2.9	*p* = 0.815
		Placebo		96.9 ± 2.5	
	Correct Passes (Deley)	CGA	98.2 ± 3.3	97.0 ± 3.3	*p* = 0.080
		Placebo		103.0 ± 1.9	
Visual Memory Test (VIM)	Correct Hits (Immediate)	CGA	84.5 ± 3.1	85.0 ± 3.0	*p* = 0.757
		Placebo		83.7 ± 3.4	
	Correct Passes (Immediate)	CGA	99.0 ± 3.4	103.9 ± 2.7	*p* = 0.835
		Placebo		103.6 ± 3.1	
	Correct Hits (Deley)	CGA	89.8 ± 3.1	91.2 ± 3.0	*p* = 0.601
		Placebo		93.0 ± 2.5	
	Correct Passes (Deley)	CGA	98.2 ± 3.3	101.1 ± 2.5	*p* = 0.435
		Placebo		102.9 ± 3.1	
Finger Tapping Test (FTT)	Right Taps Average	CGA	103.2 ± 1.7	104.8 ± 1.9	***p* = 0.017**
		Placebo		101.0 ± 2.3	
	Left Taps Average	CGA	98.0 ± 1.9	99.7 ± 1.8 ^#^	***p* = 0.049**
		Placebo		97.4 ± 1.8	
Symbol Digit Coding (SDC)	Correct Responses	CGA	109.1 ± 1.8	112.7 ± 2.6 ^#^	*p* = 0.339
		Placebo		111.4 ± 2.3	
	Errors	CGA	95.1 ± 3.1	93.2 ± 7.2	*p* = 0.935
		Placebo		96.3 ± 3.5	
Stroop Test (ST)	Simple Reaction Time	CGA	97.3 ± 2.0	98.4 ± 1.8	*p* = 0.661
		Placebo		97.7 ± 2.5	
	Complex Reaction Time	CGA	97.4 ± 2.7	94.4 ± 2.8 ^#^	*p* = 0.052
		Placebo		96.6 ± 2.5	
	Stroop Reaction Time	CGA	101.2 ± 2.5	102.0 ± 2.5	*p* = 0.293
		Placebo		100.7 ± 2.3	
	Stroop Commission Errors	CGA	89.0 ± 3.4	92.9 ± 4.4	*p* = 0.924
		Placebo		91.8 ± 2.8	
Shifting Attention Test (SAT)	Correct Responses	CGA	98.3 ± 2.4	101.5 ± 1.7	*p* = 0.721
		Placebo		101.8 ± 2.0	
	Errors	CGA	105.6 ± 1.8	106.1 ± 1.6	*p* = 0.197
		Placebo		108.0 ± 1.5	
	Correct Reaction Time	CGA	105.4 ± 2.9	109.9 ± 2.4 ^#^	*p* = 0.440
		Placebo		108.8 ± 2.4	
Continuous Performance Test (CPT)	Correct Responses	CGA	99.9 ± 2.2	99.9 ± 2.2	*p* = 0.206
		Placebo		93.6 ± 4.1	
	Commission Errors	CGA	97.4 ± 3.7	96.6 ± 2.2	*p* = 0.305
		Placebo		99.0 ± 2.2	
	Choice Reaction Time Correct	CGA	100.1 ± 2.2	98.9 ± 2.1	*p* = 0.562
		Placebo		97.7 ± 2.1	

CGA, chlorogenic acids. Data are expressed as mean ± SE, *n* = 25. ^#^ *p* < 0.05 vs. baseline, Wilcoxon signed-rank test. *p*-value; CGA vs. Placebo analyzed by Wilcoxon signed-rank test.

**Table 4:** Standardized scores of individual tests in Cognitrax.

	Group	Baseline	2W	*p*-Value
Neurocognitive Index (NCI)	CGA	98.8 ± 1.0	101.0 ± 1.0	*p* = 0.961
	Placebo		101.0 ± 1.3	
Composite Memory	CGA	87.2 ± 2.9	91.6 ± 2.9	*p* = 0.297
	Placebo		93.5 ± 2.9 ^#^	
Verbal Memory	CGA	92.8 ± 3.3	95.0 ± 2.9	*p* = 0.580
	Placebo		96.7 ± 2.3	
Visual Memory	CGA	87.6 ± 2.6	92.2 ± 2.2	*p* = 0.592
	Placebo		93.3 ± 3.0	
Psychomotor Speed	CGA	105.3 ± 1.2	108.4 ± 1.5 ^#^	***p* = 0.024**
	Placebo		105.2 ± 1.7	
Reaction Time	CGA	99.5 ± 2.8	98.5 ± 2.8	*p* = 0.796
	Placebo		98.9 ± 2.4	
Complex Attention	CGA	102.1 ± 2.3	103.4 ± 2.3	*p* = 0.345
	Placebo		104.7 ± 2.1	
Cognitive Flexibility	CGA	99.8 ± 2.1	102.6 ± 1.5	*p* = 0.381
	Placebo		103.6 ± 1.9	
Processing Speed	CGA	109.1 ± 2.0	112.6 ± 3.4 ^#^	*p* = 0.475
	Placebo		111.7 ± 2.6	
Executive Function	CGA	101.2 ± 2.1	103.5 ± 1.5	*p* = 0.360
	Placebo		104.6 ± 1.8	
Simple Attention	CGA	98.2 ± 3.2	97.6 ± 1.9	*p* = 0.802
	Placebo		97.1 ± 2.6	
Motor Speed	CGA	101.0 ± 1.7	102.5 ± 1.8	***p* = 0.013**
	Placebo		99.3 ± 2.0	

Data are expressed as mean ± SE, *n* = 25. ^#^ *p* < 0.05 vs. baseline, Wilcoxon signed-rank test. *p*-value; CGA vs. placebo analyzed by Wilcoxon signed-rank test.

## Data Availability

All data generated in this study are included in this article.
